# M2-Macrophage-Induced Chronic Inflammation Promotes Reversible Mesenchymal Stromal Cell Senescence and Reduces Their Anti-Fibrotic Properties

**DOI:** 10.3390/ijms242317089

**Published:** 2023-12-04

**Authors:** Uliana Dyachkova, Maksim Vigovskiy, Nataliya Basalova, Anastasia Efimenko, Olga Grigorieva

**Affiliations:** 1 Faculty of Medicine, Lomonosov Moscow State University, 119991 Moscow, Russia; dyachkovaud@my.msu.ru (U.D.); vigovskiy_m.a@mail.ru (M.V.); basalovana@my.msu.ru (N.B.); efimenkoay@my.msu.ru (A.E.); 2 Institute for Regenerative Medicine, Medical Research and Education Center, Lomonosov Moscow State University, 119992 Moscow, Russia

**Keywords:** chronic inflammation, multipotent mesenchymal stromal cells, macrophages, extracellular vesicles, fibroblast-to-myofibroblast differentiation, fibrosis

## Abstract

Fibrosis and the associated decline in organ functionality lead to an almost 50% mortality rate in developed countries. Multipotent mesenchymal stromal cells (MSC) were shown to suppress the development and progression of fibrosis through secreted factors including specific non-coding RNAs transferred within extracellular vesicles (EV). However, age-associated chronic inflammation can provoke MSC senescence and change secretome composition, thereby affecting their antifibrotic properties. Alternatively activated macrophages (M2-type) are key players in chronic inflammation that may interact with MSC through paracrine mechanisms and decrease their antifibrotic functions. To confirm this hypothesis, we evaluated the M2-macrophage conditioned medium (CM-M2) effect on human adipose-tissue-derived MSC senescence in vitro. We found that CM-M2, as well as a pro-senescence agent, hydrogen peroxide (H_2_O_2_), increased p21+–MSC number and secretion of IL-6 and MCP-1, which are considered main senescence-associated secretory phenotype (SASP) components. Thus, both exposures led to the senescent phenotype acquisition of MSC. EV from both CM-M2 and H_2_O_2_-exposed MSC, which showed a decreased effect on the suppression of TGFβ-induced fibroblast-to-myofibroblast differentiation compared to EV from control MSC according to αSMA level and the αSMA+–stress fiber reduction. After two weeks of subsequent cultivation under standard conditions, MSC demonstrated a decrease in senescence hallmarks and fibroblast differentiation suppression via EV. These results suggest that M2-macrophage-induced chronic inflammation can reversibly induce MSC senescence, which reduces the MSC’s ability to inhibit fibroblast-to-myofibroblast differentiation.

## 1. Introduction

Multipotent mesenchymal stromal cells (MSC) were first described in bone marrow and later in other tissues. They have the ability to proliferate and to differentiate into multiple cell types, such as adipocytes, osteocytes, and chondrocytes [1]. MSC are considered key regulators of tissue regeneration and injury repair, as well as fibrosis development in adult mammals. A substantial portion of MSC’s effects are mediated by their paracrine activity through the secretion of a wide range of bioactive factors [2]. The MSC secretome includes extracellular matrix (ECM) components, soluble factors involved in angiogenesis, wound healing, immune response, and fibrosis such as cytokines, chemokines, growth factors [3], as well as the cargo of extracellular vesicles (EV) consisting of different sizes of membrane particles (exosomes and microvesicles). Recently, we have shown that MSC-produced EV can directly suppress transforming growth factor (TGFβ)-1-induced fibroblast to myofibroblasts’ differentiation in vitro and in vivo through microRNA transfer, thus inhibiting the development of fibrosis [4,5].

It is widely known that the multicellular organisms’ regenerative potential decreases with age. MSC’s ability to proliferate and differentiate, as well as their immunomodulatory and wound healing properties, also diminish during aging and senescence [6]. At present, cell senescence can be characterized as an irreversible cell cycle arrest. Cells lose their ability to divide even under proliferative stimuli, although they remain viable and metabolically active and acquire resistance to apoptosis [7]. There are no absolutely specific senescent cell markers. The development of cell senescence causes two important changes: cell cycle arrest associated with cell cycle inhibitor accumulation (p16, p21, p53) and the senescent secretory phenotype [8]. In addition, senescent phenotype can be characterized by a number of markers: morphological changes (increased size, irregular shape), increased lysosome number and elevated beta-galactosidase activity, lamin B1 loss, etc. [9]. One of the most important changes is senescence-associated secretory phenotype (SASP) acquisition. The senescent cell secrete increases the number of growth factors, including TGFβ-1, epidermal growth factor (EGF), platelet-derived growth factor (PDGF), hepatocyte growth factor (HGF), and insulin-like growth factor 1 (IGF1), as well as cytokines/chemokines (monocyte chemoattractant protein 1-MCP1, tumor necrosis factor-alpha TNFα), protease inhibitors (plasminogen activator inhibitor-1-PAI1), interleukins (IL-1a, IL-1β, IL-6, IL-10) and matrix metalloproteinases (MMP10, MMP12), promoting chronic inflammation in tissues [10]. Senescent cells can have both autocrine and paracrine effects, inducing and worsening cell senescence within their microenvironment [10].

It is well established that chronic inflammation plays a key role in fibrosis development [11,12]. The main effector cells of chronic inflammation are macrophages [13], represented by a wide range of phenotypes [14]. M1, classically activated macrophages, protect the body against microorganisms by triggering an inflammatory response. They appear in response to lipopolysaccharide (LPS) exposure, and the cytokines interferon gamma (IFNγ) and TNFα. M1-macrophages secrete a large number of proinflammatory cytokines (TNFα, IL-1β, IL-6) and chemokines (CXCL9 and CXCL10). They also express surface markers CD80 and CD86 [15,16]. M2, alternatively activated macrophages, regulate this inflammation by secreting anti-inflammatory cytokines (IL-10, TGFβ-1), chemokines (CCL17, CCL22, CCL24), and damaged cell phagocytosis [15,16]. They appear in response to IL-4, IL-10, and IL-13 and are characterized by increased CD206 expression, a mannose receptor surface marker [17].

During damage reparation, the proinflammatory phenotype of macrophages changes to an anti-inflammatory to resolve inflammation and stimulate the regenerative processes [18]. In chronic inflammation, macrophages are constantly exposed to activation signals. Their phenotype is M2 with some features of M1 [14]. Thus, alternatively activated M2-macrophages are crucially involved in age-associated chronic inflammation [19], which causes fibrosis progression [14]. The interactions between fibroblasts (the main myofibroblasts sources in fibrosis), macrophages (the key sources of fibrosis-inducing factors) [20], and MSC (the possible regulators) play an important role in fibrosis but the details of these processes remain unclear. Our study is devoted to the possible mechanisms of paracrine interactions between M2-macrophages and MSC and their potential contribution to fibrosis regulation.

## 2. Results

### 2.1. Macrophages Differentiation and Polarization

**Figure 1:**
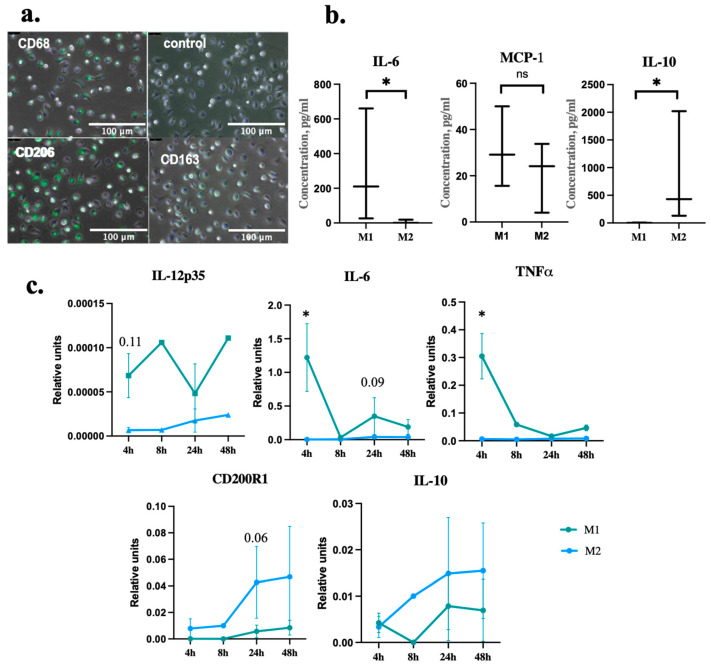
Macrophages’ differentiation and polarization. (**a**) Representative images of M2-macrophage markers’ expression (CD68, CD163, CD206-green) in IL-4-stimulated macrophages isolated from human peripheral blood. Nuclei are labeled with DAPI (blue). (**b**) IL-6, IL-10, and MCP-1 concentrations in CM from macrophages polarized in M1- and M2 types (*n* = 3). (**c**) Dynamic changes in human macrophage marker genes’ expression: M1-macrophage markers were IL-12p35, IL-6, TNFα.; M2-macrophage markers were CD200R1, IL-10. Data are presented as 10–90 percentile, line at median (*n* = 3). *p* < 0.05 (*), *p* > 0.05 (ns).

The polarization of classical M1-type macrophages was induced by a combination of LPS and IFNγ; alternative M2-type cells were obtained by IL-4 treatment for 24 h [16]. Using ICC analysis, we showed that macrophages isolated from the peripheral blood and polarized by IL-4 into M2 type expressed the pan-macrophage marker CD68 as well as M2-macrophage markers (mannose receptor CD206 and hemoglobin receptor CD163), as shown in Figure 1a. Alternatively activated macrophages produce a set of anti-inflammatory factors such as IL-10, whereas the secretion of proinflammatory cytokines such as IL-6 is reduced [21]. We analyzed the secretion of key pro- and anti-inflammatory interleukins in macrophage CM. We showed that M2-macrophages significantly increased secretion of the key anti-inflammatory interleukin IL-10 compared to M1-macrophages. Conversely, M2-macrophages secreted a reduced amount of the proinflammatory factors IL-6 and MCP-1 (CCL2), as shown in Figure 1b. We also analyzed the expression level of the genes involved in macrophage polarization by RT-PCR. As demonstrated in Figure 1c, the M1-macrophage markers expression increased with M1-macrophages (IL-12p35, TNFα, and IL-6) whereas M2-macrophages expressed M2-macrophage markers (CD200R1 and IL-10).

### 2.2. M2-Macrophages Induce MSC Senescence via Secretion of Paracrine Factors

**Figure 2:**
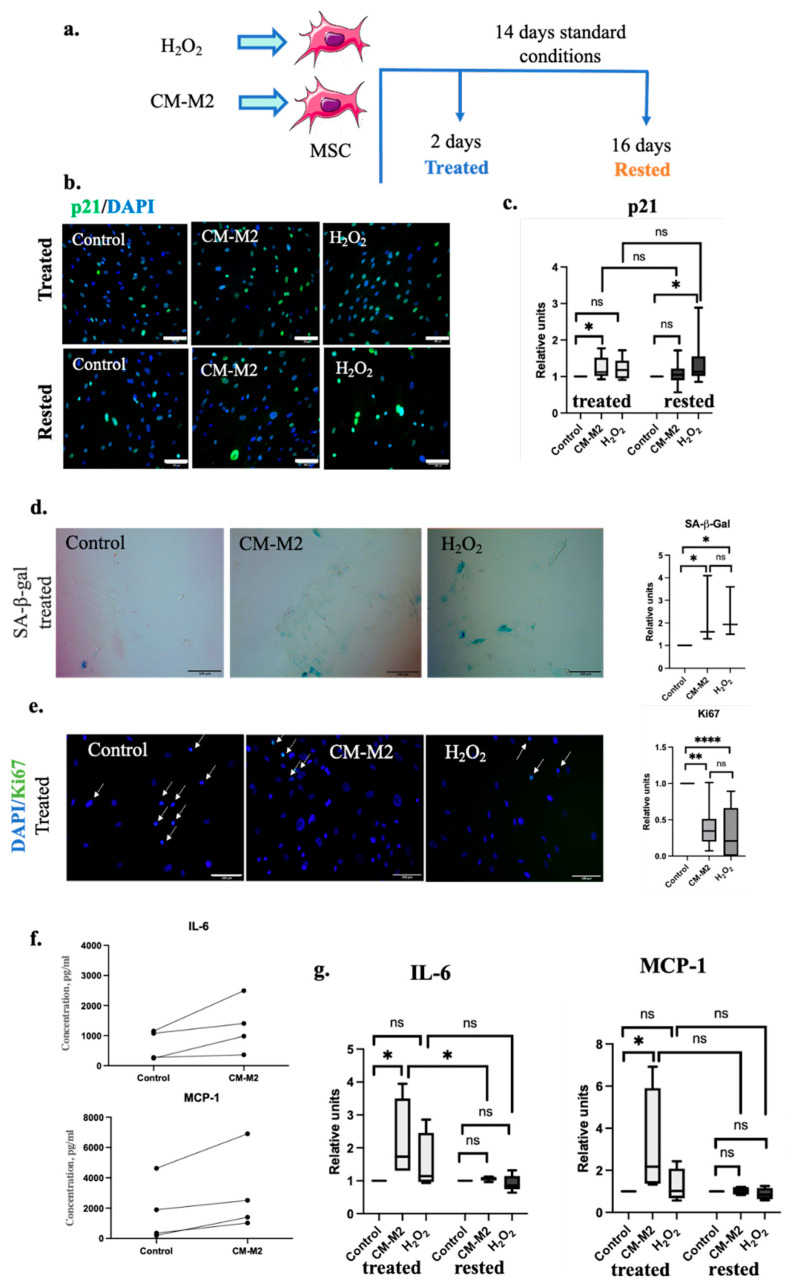
M2-macrophages induce MSC senescence through paracrine. (**a**) Experimental design. (**b**) Representative images of human p21+ MSC (p21—green). Nuclei labeled with DAPI (blue). Treated—2 days; rested—16 days (2 days of exposure, then 14 days under standard conditions). Scale bar 100 μm. (**c**) p21+ cells amount relative to control 2 days after exposure (treated); 16 days after exposure (rested). (*n* = 9). (**d**) Representative images of senescence-associated β-galactosidase. Treated—2 days of exposure. Scale bar 100 μm. SA-β-ga+ cells amount relative to control 2 days after exposure (treated) (**e**) Representative images of human Ki67+ MSC (Ki67—green). Nuclei labeled with DAPI (blue). Treated—2 days. Scale bar 100 μm. Ki67+ cells amount relative to control 2 days after exposure (treated). (**f**) IL-6 concentration (up), MCP-1 concentration in (down) conditioned by MSC medium before and after CM-M2 exposure in different donors (*n* = 4). (**g**) IL-6 and MCP-1 levels relative to control 2 days after exposure (treated), 16 days after exposure (rested). *n* = 4–5. All the data are presented as 10–90 percentile; line at median. *p* > 0.05 (ns), *p* < 0.05 (*), *p* < 0.005 (**), *p* < 0.0001 (****).

To evaluate whether the paracrine effect of M2-macrophages on MSC could promote their senescence, we obtained CM-M2, as described earlier. Hydrogen peroxide H_2_O_2_ was used as a well-known prosenescent agent for positive control [22]. MSC incubation with H_2_O_2_ for 24 h or CM-M2 for 48 h (tested immediately after exposure—treated) increased the amount of p21+ cells within the MSC population compared to the standard conditions, as shown in Figure 2b,c. We observed this effect for MSC from all donors. Further MSC cultivation under standard conditions for 14 days (in complete medium without H_2_O_2_ or CM-M2 exposure—rested) led to a significant decrease in p21+ cell number after CM-M2 exposure almost to control level. Exposure to H_2_O_2_ led to a p21+ cell percentage retention above the control level even 14 days after cultivation under standard conditions. Exposure to CM-M2 and H_2_O_2_ led to an increase in the SA-β-gal level compared to control, as shown in Figure 2d. At the same time, the expression of Ki67, the marker of cell proliferation, was decreased in cells exposed to CM-M2 and H_2_O_2_, as shown in Figure 2e.

Additionally, the incubation of MSC with CM-M2 significantly increased the concentration of crucial SASP proinflammatory and prosenescent components, such as IL-6 and MCP-1 in CM-MSC (Figure 2f). It should be noted that we observed substantial variability in the absolute values of the secreted factors in CM-MSC derived from different donors; however, their secretion increased in each case. After 2 days’ exposure of MSC to CM-M2 (treated) cells were cultured for 14 days under standard conditions (rested) and the secretion of SASP components decreased almost to the control level (Figure 2g). The incubation of MSC with H_2_O_2_ also slightly stimulated the secretion of IL-6 and MCP-1, but the observed differences were not significant. Thus, the CM-M2 effect on SASP induction in MSC was stronger compared to the standard H_2_O_2_ model of stress-induced senescence. 

### 2.3. M2-Macrophages Attenuate MSC Ability to Inhibit the Differentiation of Fibroblasts into Myofibroblasts

**Figure 3:**
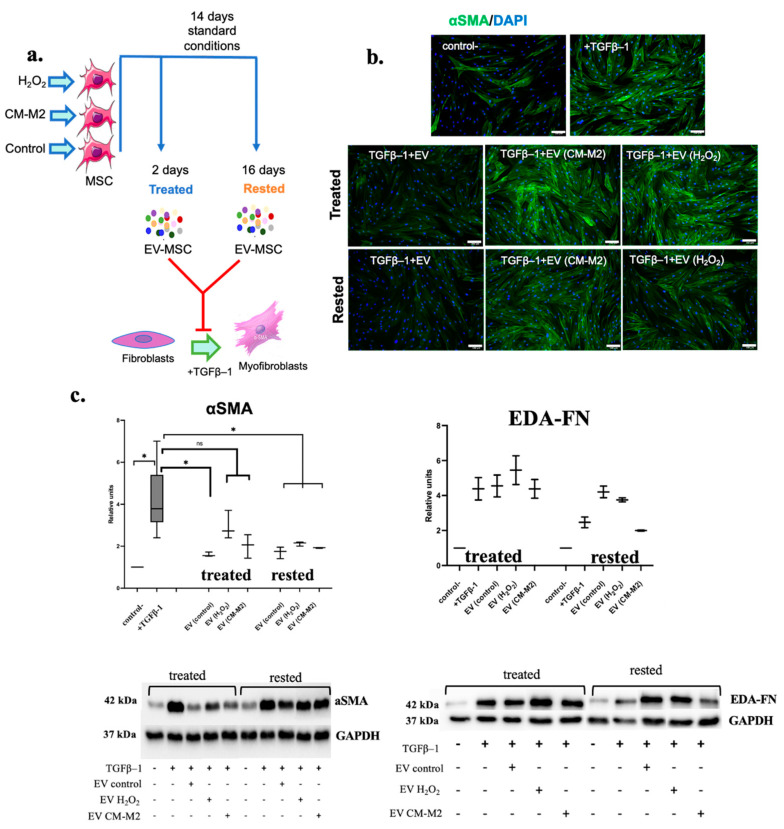
M2-macrophages attenuate MSC’s ability to inhibit fibroblasts to myofibroblasts differentiation. (**a**) Experimental design. (**b**) Representative images of alpha-actin (green) in fibroblasts without exposure (control-), under TGFβ–1 exposure (+TGFβ–1), under the simultaneous action of TGFβ–1 and EV from control MSC (EV), senescent MSC (EV CM-M2 and EV H_2_O_2_), treated and rested. Nuclei labeled with DAPI (blue). Scale bar 100 μm. (**c**) αSMA and EDA-FN protein levels in human dermal fibroblasts without exposure (control-), under TGFβ–1 exposure (+TGFβ–1), under simultaneous exposure of TGFβ–1 and EV from control MSC (EV), and under senescent MSC (EV CM-M2 and EV H_2_O_2_), measured by immunoblotting. αSMA level relative to control 2 days after exposure (treated); αSMA level relative to control 16 days after exposure (rested). Representative data obtained by immunoblotting. Data are presented as 10–90 percentile; line at median. *n* = 3. *p* > 0.05 (ns), *p* < 0.05 (*).

To evaluate if the senescence of MSC induced by CM-M2 or H_2_O_2_ affected their antifibrotic properties, we isolated an EV fraction from MSC secretome. Earlier, we demonstrated that EVs secreted by MSC were able to inhibit fibrosis by specific microRNA transfer into fibroblasts and myofibroblasts [4,5]. The EVs used in the experiments below were obtained using methods described earlier [4,5]. The particle size and number in the vesicular fraction was assessed. We showed that the particle diameter ranged from 10 to 550 nm, with a median particle size of 134.3 nm and a particle count of 0.8–1.7 × 109 particles/mL, which indicated the presence of both exosomes and microvesicles in the EV fraction of MSC secretome. Using the well-established model of TGFβ1-induced human dermal fibroblasts to myofibroblasts differentiation, we revealed that EV secreted by MSC cultured in the presence of CM-M2 or H_2_O_2_ for two days (treated) had a decreased ability to inhibit the fibroblasts for myofibroblasts differentiation, as assessed by the increased EDA-FN and αSMA expression and αSMA incorporation into stress fibers (Figure 3b). However, EV from control MSC (EV control) significantly decreased the amount of αSMA in TGFβ1-treated fibroblasts to a level comparable to the negative control; EV from MSC exposed to CM-M2 or H_2_O_2_ for two days was not so effective (Figure 3b,c). However, the subsequent cultivation of MSC under standard conditions for 14 days (rested) partially recovered the ability of EV secreted by CM-M2-treated MSC to inhibit fibroblast differentiation (Figure 3b,c). For H_2_O_2_-treated MSC, the recovery effect was not significant. 

## 3. Discussion

Macrophages play an important role in immune response regulation, being a part of the innate immunity. They determine immunity function mostly through the secretion of multiple paracrine factors. Macrophages acquire a pro-inflammatory M1 phenotype under the action of various infection- and damage-associated factors, such as LPS, TNFα, and IFNγ [14,16]. M1-macrophages secrete a wide spectrum of proinflammatory cytokines, like TNFα, IL-6, and MCP-1, supporting the acute response to damage. They are necessary for the development of inflammation and attract immune cells to the site of injury. On the other hand, macrophages can acquire so called anti-inflammatory M2 phenotype under IL-4, IL-13 and TGFβ-1 stimulation [14,16]. M2-macrophages limit the inflammatory response by secreting anti-inflammatory cytokines, including IL-10. Macrophage phenotypes differ in terms of their surface markers; thus, M1-macrophages express CD80 and CD86, whereas M2-phenotype markers are CD206 and CD163, with CD68 expressed by both phenotypes [15]. We developed an experimental protocol utilizing GM-CSF and IL-4 to stimulate human peripheral blood monocytes to differentiate into adhesive macrophages with CD68, CD206 and CD163 expression. Macrophages exhibited a high level of IL-10 secretion in combination with decreased IL-6 secretion levels, as well as a decrease in the gene expression of IL-6, IL-12p and TNFα. Thus, we demonstrated that monocytes isolated from human peripheral blood and differentiated by the developed protocol acquired the alternatively activated M2-macrophages phenotype.

There is accumulating evidence indicating the crucial role of M2-macrophages in primary aging and damage-induced cellular senescence [23,24,25]. Cellular senescence is characterized by irreversible cell cycle arrest [26]. This phenomenon is explained by cyclin-dependent kinase inhibitors’ accumulation, such as p21 and p16 [27]. In addition, there are a number of other markers that can be used to identify cell senescence. SASP acquisition is one of the most significant changes in senescent cells. This phenotype is characterized by the alterations in the paracrine secretion [28]. The SASP components are mainly proteases, proinflammatory cytokines, and chemokines such as IL-6, IL-8, MCP-1, MMPs [29]. On the one hand, SASP supports inflammation, attracts the immune cells that promote tissue repair and regeneration, and subsequently eliminates the senescent cells [30]. On the other hand, the uncontrolled release of SASP components can lead to the spread of cellular senescence. This could significantly contribute to the development of age-associated diseases, as well as the failure of regenerative processes and fibrosis progression [10].

MSC play an important role in tissue homeostasis and repair after damage. It is particularly well-known that MSC play a dual role in fibrosis regulation. Thus, MSC are considered one of the myofibroblast sources in fibrotic tissue [31]. However, MSC secretome is involved in fibrosis inhibition and resolution due to the inflammation modulation, ECM remodeling and stimulation regenerative processes [32]. One of the key mechanisms of MSC’s regulatory effects is the secretion of bioactive factors, including those within EV [33,34,35]. Recently, we revealed the antifibrotic mechanism of MSC’s involvement in the inhibition of fibrosis, mediated by specific microRNAs transferred within the secreted EV into fibroblasts, myofibroblasts and regulated fibroblasts to myofibroblast differentiation [4,5]. 

However, during aging, MSC acquire a senescent phenotype; their ability to modulate the immune response deteriorates [36], as well as their differentiation potential. Thus, the ability to adipogenic differentiation is reduced in senescent MSC [37]. MSC isolated from patients with chronic inflammation have shown signs of cellular senescence [36]. 

Considering that chronic inflammation is mainly provided by to the M2-macrophages’ exposure [14], we studied the paracrine interactions between M2-macrophages and MSC, evaluating the prosenescent effects of M2-macrophages on MSC and their potential contribution to fibrosis regulation. Indeed, we showed that the CM-M2 elevates the p21+, SA-β-gal+, and Ki67- MSC number in a manner comparable to the well-described H_2_O_2_-induced senescence model. Although there was variability in p21, Ki67 and SA-β-gal expression in cells from different donors, in all experiments, we observed a relative percentage increase within the MSC population. In addition, CM-M2 induced the SASP acquisition by MSC, realizing a significant increase in IL-6 and MCP-1 secretion levels to a greater extent compared to the H_2_O_2_ model. Taken together, we showed that alternatively activated M2-macrophages promote the acquisition of cell senescence by MSC. It is likely that M2-macrophages involved in the development of chronic inflammation stimulate the proinflammatory factor secretion by senescent MSC to maintain chronic inflammation. However, this effect was reversible in vitro by resting MSC after cultivation under standard conditions for two weeks. 

Importantly, these changes were associated with the decreased ability of MSC secretome to suppress TGFβ-1-induced fibroblasts’ differentiation into myofibroblasts through the secreted EV. We suggested that MSC’s antifibrotic properties could be significantly suppressed by paracrine interaction with M2-macrophages. Further MSC cultivation in standard conditions without exposure to CM-M2 caused signs of cell senescence loss and the restoration of their antifibrotic properties. Probably, CM-M2 induced the early senescence of MSC, which may be reversible. In vivo, the sustained chronic inflammation could drive MSC into deeper state of cellular senescence and dysfunction, but this requires further research.

## 4. Materials and Methods

### 4.1. Cell Lines

All procedures performed with tissue samples to isolate cells from patients were in accordance with the Declaration of Helsinki and approved by the Local Ethic Committee of Lomonosov Moscow State University (IRB00010587), protocol #4 (4 June 2018). All donors gave their informed consent.

### 4.2. Macrophages

Macrophages were obtained from monocytes isolated from human peripheral blood of 10 healthy donors aged from 19 to 38, both male and female. Blood was mixed with an equal volume of phosphate-buffered saline (PBS, PanEco, Moscow, Russia) without calcium and magnesium, then centrifuged on a ficoll solution gradient (1.022 g/mL) (PanEco) at 300× *g* for 30 min at +4 °C. Mononuclear cells were collected, washed with PBS solution, and placed on a CellBind adhesive-coated culture plate (Corning, Glendale, AZ, USA). After 1 h incubation, unattached lymphocytes were washed from the surface. Cells were cultivated in RPMI1640 growth medium (Gibco, Waltham, MA, USA) with 1% penicillin and streptomycin (HyClone, Logan, UT, USA), 1% GlutaMax, 1% pyruvate, 1% HEPES (all Gibco) and 10% fetal bovine serum (FBS) (BioIndustries, Haemek, Israel).

After monocyte attachment, 50 ng/mL granulocyte–macrophage colony-stimulating factor (GM-CSF) (Sci-Store, Moscow, Russia) was added for mononuclear differentiation into macrophages. On day 4, the medium was changed to complete medium with the addition of 50 ng/mL GM-CSF. On day 6, macrophage polarization was stimulated by the addition of 20 ng/mL IL-4 (Sci-Store) for M2-type or by a combination of 100 ng/mL lipopolysaccharide (LPS) (Sigma, St. Louis, MO, USA) and 20 ng/mL IFN-γ (Sigma) for M1-type in full media for 24 h.

### 4.3. Multipotent Mesenchymal Stromal Cells

Human multipotent mesenchymal stromal cells (MSC) were obtained from the biobank of the Institute for Regenerative Medicine, Medical Research and Education Center, Lomonosov Moscow State University (collection ID MSU_MSC_AD (https://human.depo.msu.ru (assessed on 1 December 2023). Seven MSC cell lines obtained from different donors were used for the experiments. Cells were cultured in AdvanceSTEM™ Mesenchymal Stem Cell Media (HyClone) containing 10% AdvanceSTEM™ Supplement (HyClone), 1% antibiotic–antimycotic solution (HyClone) at 37 °C, 5% CO_2_. Cells were passaged at 70–80% confluency using Versene solution (Paneco) and HyQTase solution (HyClone). Cells of 2–5 passages were used for the experiments.

### 4.4. Dermal Fibroblasts

Human dermal fibroblast lines were obtained from the biobank of the Institute for Regenerative Medicine, Medical Research and Education Center, Lomonosov Moscow State University (collection number MSU_FB (https://human.depo.msu.ru (assessed on 1 December 2023). Seven dermal fibroblast cell lines obtained from different donors were used for the experiments. The cells were cultured in DMEM LG with 1% GlutaMax, 1% pyruvate (all Gibco) containing 10% FBS (Gibco), 1% antibiotic–antimycotic solution (HyClone) at 37 °C, 5% CO_2_. Cells were passaged at 70–80% confluency using Versene solution (Paneco) and Trypsin-EDTA solution (Gibco). Cells of 4–15 passages were used for the experiments.

### 4.5. Conditioned Media

To obtain the conditioned medium (CM), cultured cells (macrophages or MSC) were washed three times with Hanks’ solution and serum-free medium was added (RPMI1640 for macrophages and DMEM LG for MSC). CM was collected after 48 h incubation, centrifuged at 300× *g* for 10 min to remove cell debris, and the supernatant was collected. A protease inhibitor cocktail (Roche, Basel, Switzerland) was added to the samples for enzyme immunoassay. Samples were stored at −80 °C.

To obtain EV, conditioned by MSC medium was ultrafiltrated using an Amicon filter (1000 kDa, Sigma, Italy) by centrifugation at 3500× *g* for 5–10 min. The resulting concentrated fraction contained EV. All samples were stored at −80 °C. The particle size and concentration of EV samples were analyzed via nanoparticle tracking analysis (NTA; ZetaView, Particle Metrix), Figure S1.

### 4.6. Induction of MSC Senescence

We obtained conditioned-by-M2-macrophages medium (CM-M2) to study the paracrine effect of M2-macrophages on MSC. MSC were seeded in culture plates (50,000 cells/mL); after 24 h, CM-M2 was added to cells for 48 h. H_2_O_2_ exposure was used as a reference senescence model for control [22]. In brief, 300 nM H_2_O_2_ (Sigma) was added to MSC for 24 h in complete medium. Cells were also cultivated in standard conditions for 14 days after the end of treatment with H_2_O_2_ or CM-M2 to assess the MSC senescence’s reversibility.

### 4.7. TGFβ–1-Induced Fibroblasts’ Differentiation

Human dermal fibroblasts were seeded in culture plates (20,000 cells/mL) in complete growth medium. After 24 h, the cells were incubated for 24 h in serum-free medium for deprivation, then treated with 5 ng/mL TGFβ-1 (Cell Signaling, Beverly, MA, USA) in serum-free growth medium and cultured for 4 days. The antifibrotic activity of senescent MSC secretome was evaluated by the addition of 5× EV to fibroblasts simultaneously with 5 ng/mL TGFβ–1 in serum-free growth medium for 4 days. Cells cultured in serum-free medium with or without TGFβ–1 were used as controls (negative (control-) and positive (+TGFβ–1) controls, respectively).

### 4.8. Enzyme Immunoassay

The cytokines’ concentration was measured in the CM samples by enzyme immunoassay (ELISA) according to the manufacturer’s instructions: IL-10 (550613, BD, Franklin Lakes, NJ, USA), IL-6 (550799, BD), IL-12 (551116, BD).

### 4.9. Immunocytochemical Analysis

Cells were washed with PBS, and fixed with 4% paraformaldehyde solution (Panreac, Spain) in PBS with pH 7.2–7.4 for 10 min at room temperature. Cell membranes were permeabilized with 0.2% Triton ×100 (Sigma) in PBS for 10 min at RT to detect intracellular markers. Further, cells were incubated for 1 h in 1% bovine serum albumin (BSA, Sigma) and 10% normal goat serum (Abcam, Cambridge, UK) solution at RT to block the non-specific antibody interactions. Then, the samples were then incubated with primary antibody solution or isotype control IgG as a control (Biolegend, San Diego, CA, USA) at +4 °C overnight. Polyclonal rabbit antibodies to surface macrophages markers (CD206 (ab64693, 1:1000, abcam), CD163 (ab182422, 1:1000, abcam), CD68 (ab125212, 1:1000, abcam)) were used to characterize macrophage polarization. Polyclonal rabbit antibodies to the cell-cycle inhibitor protein (one of the senescent cell markers) p21 (2947S, 1:300, Cell Signaling) and proliferation marker Ki67 (ab16667, 1:300, abcam) were used to estimate the p21+ or Ki67+ cell number. p21 and Ki67 signal was considered sufficient if it exceeded isotype control IgG signal, which was cut using threshold by ImageJ. The number of p21- and Ki67-positive nuclei was counted relative to the total nuclei number. Polyclonal murine antibodies to alpha smooth muscle actin αSMA (ab5694, 1:300, abcam) were used to analyze the differentiation of human dermal fibroblasts into myofibroblasts after TGFβ-1 and EV exposure. After primary antibodies, samples were incubated with fluorescence-labeled goat anti-rabbit or goat anti-mouse (Invitrogen, A-11001 or A11034) secondary antibodies at room temperature for 1 h. Cell nuclei were labeled with DAPI (DAKO, Glostrup, Denmark). Samples were analyzed with a Leica DM6000B fluorescent microscope equipped with a Leica DFC 360FX camera (Leica Microsystems GmbH, Wetzlar, Germany) using LasX program equipment.

### 4.10. Senescence-Associated β-Galactosidase 

SA-β-gal staining was performed according to the manufacturer’s instructions using the Senescence β-Galactosidase Staining Kit (9860, Cell Signaling, Beverly, MA, USA).

### 4.11. Protein Electrophoresis and Immunoblotting

Human dermal fibroblasts were lysed on ice in 2× Laemmli sample buffer (Bio-Rad, Hercules, CA, USA). Protein concentration was measured using a commercial DC Protein Assay (Bio-Rad, Hercules, CA, USA) according to the manufacturer’s instructions. Cell lysate proteins were separated by denaturing phoresis with 20% SDS (Sigma). The standard commercial mixture of pre-stained proteins PageRuler™ Plus (Invitrogen) was used to determine the molecular weights of proteins. After electrophoresis, proteins were transferred from the gel to an Amersham Hybond-P PVDF membrane (GE Healthcare, Chalfont Saint Giles, UK) by electroblotting at 100 V for 60 min. Non-specific antibody interactions were blocked in TBS/Tween containing 5% milk for 1 h at room temperature. Immunoblotting was performed using unlabeled monoclonal antibodies specifically binding to αSMA (Abcam, 1: 500, ab5694), EDA-FN (ab6328, 1:1000, abcam), and GAPDH (2118, Cell Signaling, 1:1000) for protein amount normalization, overnight at +4 °C, followed by incubation with 1:3000 s antibodies (rabbit goat IgG antibodies or mouse rabbit IgG antibodies) conjugated with horseradish peroxidase (Sigma, USA) for 1 h at room temperature. Proteins bound to antibodies were visualized using a chemiluminescent substrate (Clarity™ Western ECL Substrate, Bio-Rad, Hercules, CA, USA). Chemiluminescence detection was performed using the ChemiDoc Imaging System (Bio-Rad, Hercules, CA, USA). Densitometric analysis was performed using ImageLab software (Version 6.0.1).

### 4.12. Real-Time Polymerase Chain Reaction (PCR-RT) with Reverse Transcription

For PCR-RT, macrophages were lysed in RLT buffer (Qiagen, Hilden, Germany) with 1% beta-mercaptoethanol (Sigma). Total RNA was isolated from cell lysates using the RNeasy Mini Kit (Qiagen, Germany) according to the manufacturer’s instructions. The concentration and purity of the isolated total RNA were determined using a NanoDrop 1000 spectrophotometer (Thermo Scientific, Waltham, MA, USA) using the original ND-1000 V 3.7.1 software (Thermo Scientific). Samples with absorbance ratios at 260 and 280 nm (A260/280) from 1.9 to 2.1 were used for further analysis. cDNA synthesis was performed using the MMLV RT kit (Eurogen, Moscow, Russia) according to the manufacturer’s instructions. Amplification was performed using a Nexus Mastercycler^®^ gradient (Eppendorf, Hamburg, Germany). Quantitative real-time PCR was performed using the qPCRmix-HS SYBR + LowROX kit (Eurogen) according to the manufacturer’s protocols on a QuantStudio™ 5 Real-Time PCR System (Applied Biosystems, Waltham, MA, USA). The primer sequences that were used are shown in Table 1.

### 4.13. Statistical Analysis

Experimental data are presented as 10–90 percentile; line at meridian. Statistical processing was conducted using GraphPad Prism 9.0 software. Mann–Whitney nonparametric test was used to check the statistical significance of data between experimental and control groups. Differences were considered statistically significant when *p* < 0.05.

## 5. Conclusions

Thus, alternatively activated M2-macrophages induce MSC cellular senescence and reduce the ability of EV produced by MSC to suppress TGFβ-1-induced fibroblasts into myofibroblasts’ differentiation. This process could significantly contribute to the development and progression of fibrosis. However, these effects may be reversible after the termination of prosenescent factor’s exposure to MSC. We suggest that a short exposure to the cytokines involved in chronic inflammation mediated by M2-macrophages may lead to the development of early but not deep cellular senescence. This indicates the importance of studying the interactions between macrophages and MSC during the development of fibrosis to clarify the mechanisms of MSC senescence under long-term chronic inflammation and the associated changes in their antifibrotic properties, as well as the search for approaches to control these processes.

## Figures and Tables

**Table 1:** Human gene primer sequences.

Gene	Forward 5′→3′	Reverse 5′→3′
36b4	GCTGCTGCCCGTGCTGGTG	TGGTGCCCCTGGAGATTTTAGTGG
IL-12p35	GATGGCCCTGTGCCTTAGT	TCAAGGGAGGATTTTTGTG
CD200R1	GGAGGATGAAATGCAGCCCTA	CTCAGATGCCTTCACCTTGTT
TNFα	GAGGCCAAGCCCTGGTAT	CGGGCCGATTGATCTCAGC
IL-6	ACTCACCTCTTCAGAACGAATTG	CCATCTTTGGAAGGTTCAGGTTG
IL-10	GACTTTAAGGGTTACCTGGGTTG	TCACATGCGCCTTGATGTCTG

## Data Availability

The study did not report any data.

## Supplementary Materials

The following supporting information can be downloaded at: https://www.mdpi.com/article/10.3390/ijms242317089/s1.
