# Supplementary material for: M2-Macrophage-Induced Chronic Inflammation Promotes Reversible Mesenchymal Stromal Cell Senescence and Reduces Their Anti-Fibrotic Properties
Source: Int J Mol Sci. 2023 Dec 4;24(23):17089. doi: 10.3390/ijms242317089 (PMC10707628; doi:10.3390/ijms242317089)
Supplement: Supplementary file 1 [file ijms-24-17089-s001.zip › ijms-2672731-supplementary.pdf]

Supplement 1.

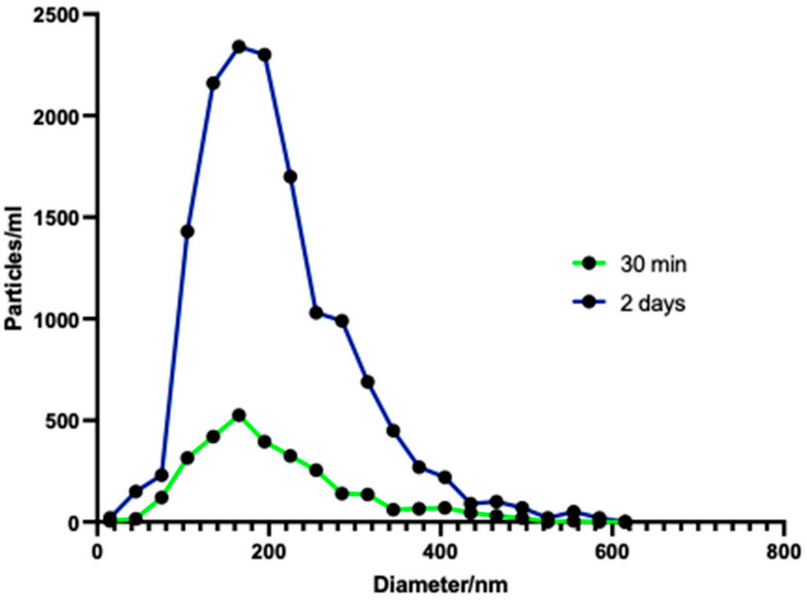

Characteristic	EVs MSCs
Concentration, particles/ml	$4,2x10^8$
Peak size, nm	174,4
Mean size, nm	211,3

Figure S1. MSC-EV characteristics by NTA.
